# Improving the Solubility and Digestibility of Potato Protein with an Online Ultrasound-Assisted PH Shifting Treatment at Medium Temperature

**DOI:** 10.3390/foods9121908

**Published:** 2020-12-20

**Authors:** Chao Mao, Juan Wu, Xiangzhi Zhang, Fengping Ma, Yu Cheng

**Affiliations:** 1 School of Food and Biological Engineering, Jiangsu University, 301 Xuefu Road, Zhenjiang 212013, China; 2221718003@stmail.ujs.edu.cn (C.M.); wujuan@ujs.edu.cn (J.W.); 3170905019@stmail.ujs.edu.cn (X.Z.); 3170905010@stmail.ujs.edu.cn (F.M.); 2 Institute of Food Physical Processing, Jiangsu University, 301 Xuefu Road, Zhenjiang 212013, China; 3 Jiangsu Provincial Key Laboratory for Food Physical Processing, 301 Xuefu Road, Zhenjiang 212013, China

**Keywords:** potato protein, ultrasound, pH shifting, solubility, digestion

## Abstract

Ultrasonic (US) treatment was combined with pH shifting (pHS) and mild thermal (40 °C) (T40) treatment (US/T40/pHS) to improve the solubility of potato protein. The effects of the ultrasonication frequency, ultrasonication time, and incorporation sequence on the solubility of potato protein were investigated. The results showed that online US/T40/pHS treatment resulted in higher solubility of potato protein and enhanced free amino group release during in vitro digestion. The solubility of potato protein treated with online US/T40/pHS at a mono-frequency of 40 kHz for 15 min increased by 1.73 times compared with the control (*p* < 0.05). The digestibility rate increased by 16.0% and 30.8% during gastric and intestinal digestion, respectively, compared with the control (*p* < 0.05). It was demonstrated that online US/T40/pHS treatment significantly changed the secondary and tertiary structures of potato protein according to the results of circular dichroism and internal fluorescence. SDS-PAGE, particle size, and atomic force microscopy (AFM) showed that structural changes led to the formation of large soluble aggregates. The results suggested that the improvement in the solubility and digestibility of potato protein treated with online US/T40/pHS may be due to the formation of large soluble aggregates, which are more hydrophilic and sensitive to digestive enzymes.

## 1. Introduction

Potato protein is considered to be a high-quality plant protein due to its good amino acid composition [1]. Its amino acid composition resembles that of egg protein [2]. Generally, potato protein is a by-product of the starch industry [3]. The high-intensity heat treatment in the recycling process decreases the solubility of potato protein, which limits its application [4]. Therefore, it is interesting to explore methods that can improve the solubility of potato protein.

pH shifting treatment has been shown to be an efficient way to improve the solubility of plant proteins, such as soy protein [5,6,7], pea protein [8,9], and hemp seed protein [10]. pH shifting treatment is a method that adjusts the pH of an environment to an extremely acidic or alkaline value, which is followed by adjusting the pH back to neutral. The unfolding and refolding of a protein structure during pH shifting treatment can improve the functional characteristics of the protein [5]. Optimized pH shifting treatment of plant proteins, including soy protein and pea protein, is usually conducted at an alkaline pH of 12.0 for 1 h [8,11]. However, the efficiency of pH shifting treatment on the solubility of potato protein is not clear.

Thermal processes have been shown to enhance the efficiency of pH shifting treatment on the improvement of protein solubility [10,12]. Wang et al. demonstrated that the temperature of the thermal process had a large effect on the formation of lysinoalanine (LAL) [10]. An extreme alkaline pH shifting treatment at temperatures above 40 °C sharply increased the formation of LAL in hemp protein [10]. The formation of LAL results in the loss of amino acid residues, such as Lys [13]. It is necessary to control the thermal process at mild temperatures during pH shifting treatment.

The physical and chemical forces generated from the cavitation effect of ultrasound can induce structural changes in proteins and disperse protein aggregates [14,15], which might increase interactions between water and protein molecules. Therefore, ultrasound treatment has been shown to enhance the solubility of proteins in several studies [8,16,17]. Furthermore, it was shown that the efficiency of the pH shifting processing on pea protein [8], soy protein [6], and barley protein isolate [18] was enhanced when combined with ultrasound treatment.

Although combined pH shifting with mild thermal or ultrasonic treatment has been able to improve protein solubility, few studies have investigated the effect of combining these three treatments on protein solubility. Additionally, the effect of the combination of these three treatments on the solubility of potato protein is unknown.

Therefore, the purpose of this study was to determine the effect of an ultrasound-assisted pH shifting treatment at a mild temperature on the solubility and digestibility of potato protein. Circular dichroism, fluorescence, atomic force microscopy (AFM), and SDS-PAGE were used to indicate the role of US/T40/pHS in improving the solubility and digestibility of potato protein.

## 2. Materials and Methods

### 2.1. Materials

Fresh potato was purchased from a local supermarket. 5,5’-dithiobis(2-nitrobenzoic acid) (DTNB), 8-aniline-1-naphthalenesulfonic acid (ANS), 2,4,6-trinitrobenzenesulfonic acid (TNBS), β-mercaptoethanol (β-ME), N-ethylmaleimid (NEM), pepsin, and pancreatin were purchased from Sigma Aldrich (St. Louis, MO, USA). All other reagents used in the experiment were of analytical grade.

### 2.2. Preparation of Potato Protein

Potato protein was prepared according to the method of van Koningsveld et al. [19] with some modifications. Fresh potatoes were washed and cut into small pieces. The potato pieces were suspended immediately in a sodium sulfite solution (0.12 wt %) at a ratio of 1/4 (kg/L) at 4 °C and then crushed with a grinder. Potato juice was settled for 10 min, and the supernatant was filtered through double-layer gauze. The filtration juice was centrifuged at 12,250× *g* for 15 min at 4 °C. The pH of the collected supernatant was adjusted to 8.0 with 2 M NaOH and stirred for 2 h in an ice bath. After filtration and centrifugation, the supernatant was adjusted to pH 4.0 using 1 M HCl and stirred for 1 h in an ice bath to precipitate potato protein. Then, the potato protein suspensions were centrifuged at 12,250× *g* for 15 min at 4 °C. The precipitates were collected and removed with distilled water. The potato protein solution was adjusted to pH 7.0 and lyophilized. The potato protein powder was kept in a desiccator until use.

### 2.3. Ultrasound-Assisted pH Shifting Treatment of Potato Protein

**Figure 1:**
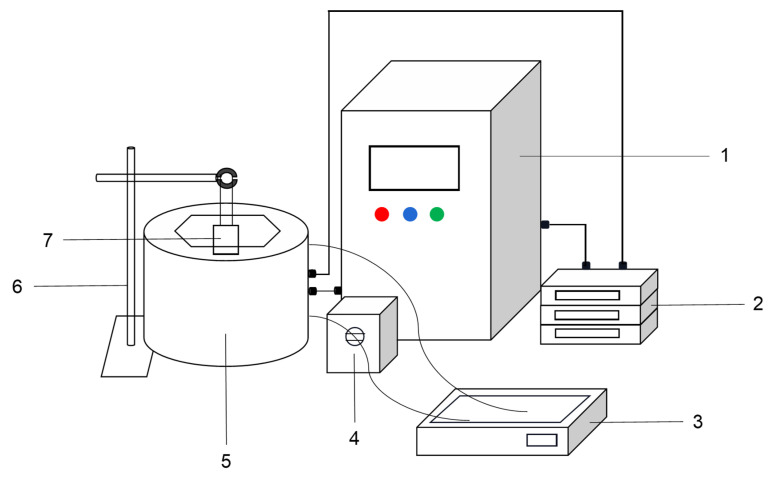
Divergent tri-frequency ultrasound equipment (Note: 1. Control panel, 2. Ultrasonic generator, 3. Constant temperature water bath pot, 4. Peristaltic pump, 5. Reactor, 6. Iron rack table, 7. Potato protein solution).

Potato protein (20 mg/mL) was dissolved in 10 mM phosphate buffer (PB) (pH 7.0) and stirred for 1 h with a magnetic stirrer at 40 °C. The potato protein suspensions were adjusted to pH 12.0 with 2 M NaOH. Ultrasound treatment was incorporated into the pH shifting treatment of potato protein with tri-frequency ultrasound equipment developed by our group and manufactured by Meibo Biotechnology Co., Ltd. (Zhenjiang, China) (Figure 1). After being maintained at pH 12.0 for 1 h, the pH of the suspensions was adjusted back to pH 7.0 with 2 M HCl. The supernatant was collected after centrifugation (10,000× *g*, 10 min) and stored in a refrigerator at −20 °C before use.

The effects of the ultrasonication frequency (triple frequencies of 20/28/40 and 20/40/60 kHz; dual frequencies of 20/28, 20/40, 20/60, 28/40, and 40/60 kHz; and mono frequencies of 20, 28, 40, and 60 kHz), the incorporation time of ultrasound (pre, post- or online pH shifting), and ultrasonication time (0, 2, 5, 10, 20, and 30 min) on the solubility of potato protein were assessed. The incorporation time of ultrasound by pre, post, or online meant that ultrasound was conducted before, after, or during pH shifting at 40 °C All ultrasound treatments were conducted in the synchronous mode with pulse on and off times of 10 and 2 s, respectively. The input energy density used for all samples was controlled at the same level of 37.5 kJ/L.

### 2.4. Solubility

The protein concentration in solution was measured by the Biuret method using bovine serum albumin as the standard [11]. The total protein content in potato protein was measured by the Kjeldahl method. Protein solubility was expressed as follows.

(1) $$\mathrm{protein}\;\mathrm{solubility}\left(\%\right)=\frac{\mathrm{protein}\;\mathrm{concentration}\;\mathrm{of}\;\mathrm{solution}\;}{\mathrm{Total}\;\mathrm{protein}\;\mathrm{concentration}}\text{×}100\%$$

### 2.5. In Vitro Digestion

In vitro stomach and intestinal digestion of potato protein was performed following the method described by Minekus et al. [20] with some modifications. The same volume of the potato protein solution (20 mL) was mixed with simulated gastric fluid (SGF) at 37 °C. The mixture was adjusted to pH 3.0. Porcine pepsin was added to the mixture at a final concentration of 2000 U/mL. After 2 h of gastric digestion, the pH of the gastric digestive juice was adjusted to 7.0. Then, 40 mL of the simulated intestinal fluid (SIF) solution was added to the gastric digestive juice. Pancreatin and bile salts were added at final concentrations of 100 U/mL and 10 mM, respectively. Gastric or intestinal digestive juice (20 μL) was withdrawn at 0, 2, 4, 6, 8, 10, 20, 40, 60, and 120 min to determine the release of free amino groups. Intestinal digestion was performed 2 h at 37 °C. The enzymes in these solutions were inactivated by liquid nitrogen.

The free amino acid content was determined using the TNBS method, which was slightly modified according to Spellman [21]. The first-order kinetic equation was used to fit the free amino release curve during the digestion process as follows:

(2) $${y-y}_{0}{=A(1-e}^{-\mathrm{kx}})$$

where *x* is the digestion time (min), *y* is the concentration of free amino groups released at time *x*, *y_0_* is the concentration of free amino groups (mM) at 0 min, A is the amount of free amino groups released at the end of the digestion (mM), and K is the rate constant (min^−1^) of the first-order kinetic reaction.

### 2.6. Free and Total Sulfhydryl Contents

The contents of free sulfhydryl and total sulfhydryl in the potato protein solution were determined according to Patrick et al.’s [22] specifications. The potato protein solution (1 mL) was mixed with tris-glycine buffer (86 mM tris, 90 mM Gly, 4 mM EDTA-Na_2_, pH 8.0) containing 8 M urea, followed by the addition of 50 μL DTNB solution. The mixture was incubated at 25 °C for 30 min. When the free sulfhydryl content was determined, buffer without urea was used. The absorbance of the mixture was measured at 412 nm using a spectrophotometer (UV-2008 Spectrometer, Unico Scientific Instrument Co., Ltd., Shanghai, China):

(3) $$\mathrm{SH}\mu \mathrm{mol}/g=\frac{73{.53\text{×}A}_{412}\text{×}D}{C}$$

where *D* is the dilution factor of the samples and *C* is the initial protein content (mg/mL).

### 2.7. Surface Hydrophobicity

Surface hydrophobicity (H_0_) was measured according to the method described by Wang et al. [10] with slight modification. The potato protein solution was diluted 10, 20, 30, 40, and 50 times with 10 mM PB (pH 7.0). The diluted solution (4 mL) was mixed with 20 µL of ANS solution (8 mM) and incubated for 3 min. The fluorescence intensity of potato protein was measured (Cary Eclipse, Varian Company, Palo Alto, CA, USA) at excitation and emission wavelengths of 290 nm and 344 nm, respectively. The initial slope of the fluorescence intensity versus protein concentration calculated by linear regression analysis was identified as the surface hydrophobicity of the samples.

### 2.8. Intrinsic Fluorescence

The intrinsic fluorescence of the potato protein solutions (2 mg/mL) was recorded at wavelengths of 290 to 500 nm with an excitation wavelength of 290 nm using a Cary Eclipse fluorescence spectrophotometer (Cary Eclipse, Varian Company, Palo Alto, CA, USA).

### 2.9. Circular Dichroism (CD) Spectrum

The CD of the potato protein samples (0.02 mg/mL) was recorded in the wavelength range of 190–250 nm with a circular dichroism spectrometer (J-815, JASCO Corporation, and Tokyo, Japan). The average of three scans at a scanning speed of 100 nm/min and a scanning step of 0.5 nm was determined. The data are expressed by the average residue ellipticity ([θ]×10^4^ (deg•cm^2^•dmol^−1^)), and the relative content of the secondary structure of the protein was calculated using the estimation program provided with the Jasco spectropolarimeter.

### 2.10. Electrophoresis

Sodium dodecyl sulfate-polyacrylamide gel electrophoresis (SDS-PAGE) was used to analyze the potato protein samples as described by Wang et al. [23] with slight modifications. The supernatant of the potato protein samples was mixed with an equal volume of sample buffer with or without 5% β-ME before boiling for 5 min. NEM (1 mM) was added to the samples without β-ME to disrupt the formation of disulfide bonds induced by heating. The acrylamide concentrations of the stacking and resolving gels were 5% and 15%, respectively. After electrophoresis, the gels were stained with 0.25% Coomassie Brilliant Blue R250 for 1 h and destained with a solution containing 45% (v/v) acetic acid and 10% (v/v) methanol until the background was clear.

### 2.11. Particle Size and Zeta Potential

The particle sizes and the ζ potentials of soluble potato protein were determined by an Anton-Paar Litesizer 500 instrument (Anton-Paar, Graz, Austria) at 25 °C. The refractive indices of the material and solvent were 1.45 and 1.33, respectively.

### 2.12. Atomic Force Microscope (AFM)

Eight microliters of the potato protein supernatant (0.04 mg/mL) were placed on mica flakes and dried in air overnight. Then, the mica pieces were placed on the sample stage (Multimode 8, Bruker Company, Ettlingen, Germany) and observed with a probe (Olympus AC160TS, Bruker Company, Ettlingen, Germany) with a strength of 26 N/m. The sweep frequency was set to 256 Hz.

### 2.13. Statistical Analysis

All experiments were set up with at least three parallel samples and three replicates. Each replication was performed on different days. Data were analyzed using SPSS 16.0 (SPSS Inc., Chicago, IL, USA) with one-way ANOVA. Significant differences in data (*p* < 0.05) were analyzed using Tukey’s multiple comparison analysis. Graphs were plotted with SigmaPlot 10 (Systat Software Inc., Chicago, IL, USA).

## 3. Results

### 3.1. Solubility

#### 3.1.1. Effect of Different Modification Methods on Potato Protein Solubility

**Figure 2:**
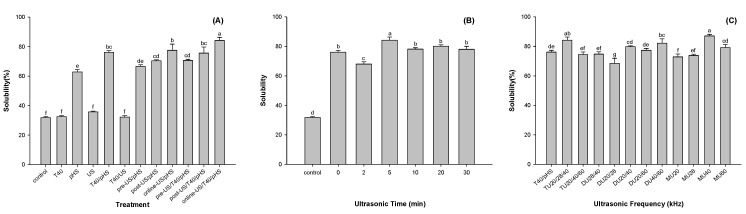
Effects of a combination of different modification methods (**A**) of mild thermal process (T40), ultrasound (US: TU20/28/40 kHz) and pH shift (pHS), ultrasonic time (**B**) and ultrasonic frequency (**C**) on the solubility of potato protein. (TU: triple-frequency, DU: dual-frequency, MFU: mono-frequency ultrasounds). Means with different letters (a–g) differ significantly (*p* < 0.05).

Solubility is regarded as the most practical indicator of the functional properties of proteins [8]. The effects of different modification methods on the solubility of potato protein are shown in Figure 2A. Compared with the control, pH shifting treatment (pHS) increased the solubility of potato protein by 31.0% (*p* < 0.05). Although treatment with ultrasound (US) or thermal processes at 40 °C (T40) did not enhance the solubility of potato protein, the combination of pH shifting with ultrasound (US/pHS) or a thermal process of 40 °C (T40/pHS) could enhance the efficiency of pHS. The solubilities of the potato proteins treated with T40/pHS and online US/pHS were 1.21 and 1.23 times higher than that of potato proteins treated with the pH shifting treatment, respectively (*p* < 0.05). As both ultrasonic and pH shifting treatments can lead to structural changes in proteins, the incorporation sequence of ultrasound into pH shifting might result in different effects on the protein structure, which might affect protein solubility. Compared with the solubility of the potato protein treated with pre- or post-US/pHS, that of online US/pHS increased by 16.4% and 10.1% (*p* < 0.05), respectively. The structure of potato protein was folded before or after pH shifting, and pre- or post-ultrasound treatment changed little on the structure of the protein [17], while the structure of potato protein was unfolded at pH12. The expansion of protein structure was more intense under the same energy input.

Interestingly, the combination of online US and T40 with pHS (online US/T40/pHS) resulted in a higher solubility of potato protein than online US/pHS and T40/pHS. The solubility of potato protein treated with online US/T40/pHS increased by 10.6% and 8.5% (*p* < 0.05) compared with the solubility of potato protein treated with T40/pHS and online US/pHS, respectively. Thus, potato protein treated with online US/T40/pHS was used in the following trials.

#### 3.1.2. Effect of Ultrasonication Time on the Solubility of Potato Protein

As shown in Figure 2B, the ultrasonication time had a significant effect on the solubility of potato protein. At an ultrasonication time of 2 min, the solubility of potato protein was lower than that of the samples without US treatment. It seemed that low ultrasonic energy promoted the formation of insoluble aggregates of potato protein. Increasing the energy input was able to unfold and disperse potato protein. The highest solubility was observed at an ultrasound time of 5 min. However, extending the ultrasonication time might result in refolding of the potato protein and decrease its solubility. Our results were consistent with other studies showing that a higher input energy led to the formation of aggregates and loss of solubility [24,25].

#### 3.1.3. Effect of Ultrasonication Frequency on the Solubility of Potato Protein

The effects of triple-frequency (TU), dual-frequency (DU), and mono-frequency (MU) ultrasound on the solubility of potato protein are shown in Figure 2C. Among them, TU20/28/40, DU40/60, and MU40 kHz significantly improved the solubility of potato protein (*p* < 0.05). The solubility of potato protein treated with online US/T40/pHS at frequencies of TU20/28/40, DU40/60, and MU40 kHz increased by 10.6%, 7.9%, and 14.4%, respectively, compared with T40/pHS. This result suggested that the frequency of 40 kHz might be the core frequency to improve the solubility of potato protein. The combination of different frequencies might interfere with each other and produce different effects on the functional properties of the protein [26]. Compared with the MU treatment, the working time of the TU or DU treatment was shorter. Thus, TU20/28/40 kHz, DU40/60 kHz, and MU40 kHz were selected for subsequent trials.

### 3.2. Effect of Online US/T40/pHS Treatment on Potato Protein Digestion

**Figure 3:**
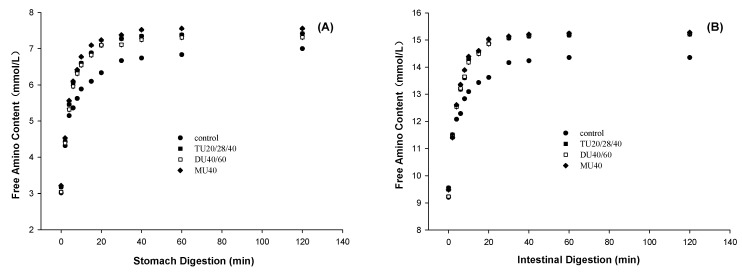
Effects of potato protein treated with online ultrasonic (US) treatment combined with pH shifting (pHS) and mild thermal (40 °C) (T40) treatment (US/T40/pHS) at the frequency of TU20/28/40, DU40/60, and MU40 kHz on the release of free amino group of potato protein during stomach (**A**) and intestinal (**B**) digestion.

The digestibility of protein was characterized by measuring the content of free amino groups during a simulated gastrointestinal digestion. The effect of online US/T40/pHS treatment on the release of free amino groups from potato protein during gastric and intestinal digestion is shown in Figure 3A,B, respectively. The first-order kinetic equation (Equation (2)) was used to fit the curves to describe the free amino acid release during digestion. The content of the final free amino group released A and digestion rate constant K are shown in Table 1. During gastric digestion, compared with the control, the A values of TU20/28/40, DU40/60, and MU40 kHz increased by 12.1%, 10.9%, and 14.1% (*p* < 0.05), while the K value increased by 20.0%, 24.4%, and 16.0% (*p* < 0.05), respectively. The results of intestinal digestion were similar to those of gastric digestion. During intestinal digestion, compared with the control, the A values of TU20/28/40, DU40/60, and MU40 kHz increased by 16.6%, 19.2%, and 19.2% (*p* < 0.05), respectively, while the K value increased by 25.8%, 30.8%, and 30.8% (*p* < 0.05), respectively. These results showed that online US/T40/pHS treatment improved the digestibility rate and digestibility of potato protein. There were no significant differences in protein digestibility among TU20/28/40 kHz, DU40/60 kHz, and MU40 kHz.

### 3.3. Effect of Online Us/t40/pHS Treatment on the Structural Characteristics of Potato Protein.

#### 3.3.1. Free and Total Sulfhydryl Contents

**Figure 4:**
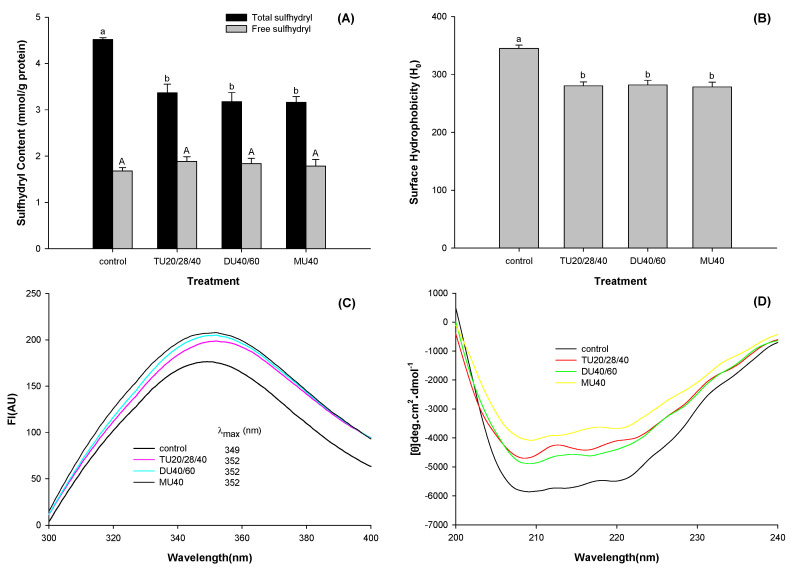
Effects of online-US/T40/pHS treatment at the frequency of TU20/28/40, DU40/60, and MU40 kHz on sulfhydryl content (**A**), surface hydrophobicity (**B**), intrinsic fluorescence (**C**), and circular dichroism (**D**) of potato protein. Means with different letters (a–b or A–B) differ significantly (*p* < 0.05).

The solubility of a protein is directly related to the structure of the protein, and the sulfhydryl content is a significant indicator of protein structure. As shown in Figure 4A, compared with the control, the total sulfhydryl content of potato protein treated with TU20/28/40, DU40/60, and MU40 kHz decreased by 25.4%, 29.7%, and 30.0%, respectively (*p* < 0.05), while the free sulfhydryl content only slightly changed (*p* > 0.05). Under alkaline conditions, unfolded protein may result in exposure to sulfhydryl groups. These sulfhydryl groups can be oxidized and converted into sulfhydryl compounds (-S-OH) and disulfide bonds (-S-S) [27], which can decrease the total sulfhydryl group content. On the other hand, the structure of the protein refolded when the pH of the solution was adjusted back to neutral. Some of the sulfhydryl groups were wrapped into the protein molecule again. Therefore, the content of free sulfhydryl had no significant change (*p* > 0.05) [7].

#### 3.3.2. Surface Hydrophobicity

Hydrophobic interactions of proteins are among the main factors that affect the solubility of proteins. As displayed in Figure 4B, online US/T40/pHS treatment decreased the surface hydrophobicity (H_0_) of potato protein (*p* < 0.05). Compared with the control, the H_0_ value of potato protein treated with TU20/28/40, DU40/60, and MU40 kHz was reduced by 18.7%, 18.3%, and 19.3% (*p* < 0.05), respectively. This reduction might be due to the formation of large soluble potato protein aggregates and the entrapment of hydrophobic groups in those aggregates.

#### 3.3.3. Intrinsic Fluorescence

The change in the endogenous fluorescence spectrum of a protein can reflect the change in its tertiary structure. As shown in Figure 4C, compared with the control, the fluorescence intensity (FI) of potato protein treated with TU20/28/40, DU40/60, and MU40 kHz increased by 13.9%, 16.2%, and 17.9% (*p* < 0.05), respectively. Moreover, the maximum emission wavelengths (λ_max_) of potato protein treated with TU20/28/40, DU40/60, and MU40 kHz all indicated a redshift from 349 to 352 nm.

#### 3.3.4. Circular Dichroism Spectra Measurement

The secondary structure of potato protein treated with online US/T40/pHS was assessed using CD spectroscopy, as shown in Figure 4D. The CD spectrum of potato protein exhibited two negative bands at around 208 and 222 nm, which represent α-helixes [28]. After TU20/28/40, DU40/60, and MU40 kHz treatment, the average residue elasticity of the negative peaks was significantly reduced, which suggested that online US/T40/pHS treatment reduced this form of secondary structure of potato protein.

To analyze the secondary structure components of potato protein intuitively, CD Pro software was used to calculate their content. As displayed in Table 2, compared with the control, the α-helix content of potato protein treated with TU20/28/40, DU40/60, and MU40 kHz decreased by 54.5%, 53.4%, and 56.7% (*p* < 0.05), respectively, while the β-sheet content increased by 40.2%, 39.6%, and 34.8% (*p* < 0.05), respectively. The contents of β-turns and random coils changed little (*p* > 0.05). Most of the secondary structures of potato protein were still retained according to the curves shown in Figure 4D and the data presented in Table 1. This result suggested that the potato protein was in a molten globule state with incomplete denaturation [29].

#### 3.3.5. Electrophoresis

The main components of potato protein are patatin (40 kDa) and protease inhibitors (15–25 kDa), which account for approximately 40% and 50% of the potato protein components, respectively [1].

**Figure 5:**
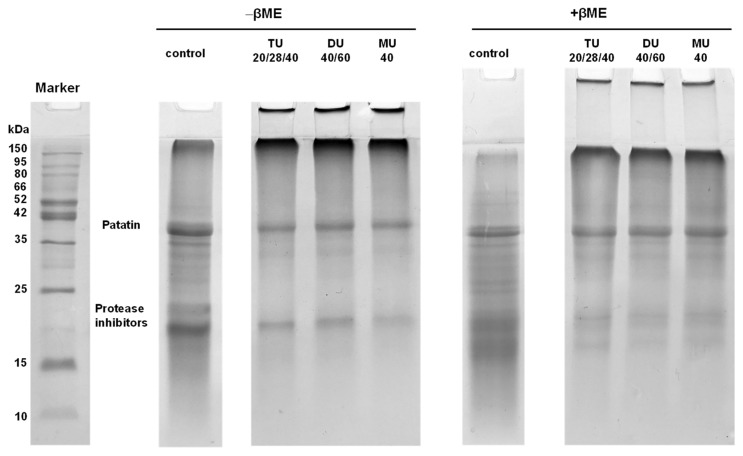
Reducing β-mercaptoethanol (+βME) and non-reducing (-βME) SDS-PAGE of potato protein treated with online-US/T40/pHS at the frequency of TU20/28/40, DU40/60, and MU40 kHz.

SDS-PAGE was used to analyze the changes in various components in potato protein treated with online US/T40/pHS. As displayed in Figure 5, the band intensities of patatin and protease inhibitor in modified potato protein were lower than those in the control. A large number of soluble aggregates were found at the top of the separation gel and loading port. After adding β-ME to the samples, some of the large soluble aggregates did not disappear. As noncovalent and disulfide bonds were interrupted by SDS and β-ME, respectively, it seemed that other covalent bonds might be formed in those soluble aggregates, such as the Schiff base and dityrosine [10]. Similar results have been shown by other researchers [10,11,17].

**Figure 6:**
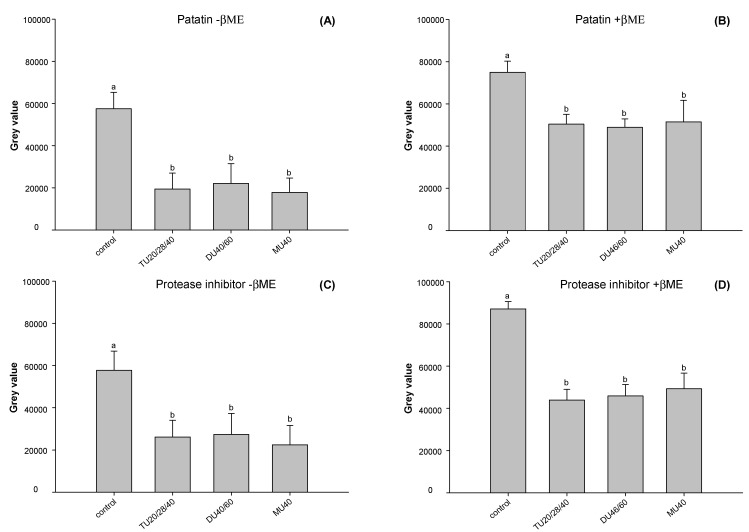
The band intensity of patatin (**A**,**B**) and protease inhibitor (**C**,**D**) of potato protein in reducing (+βME) and non-reducing (–βME) SDS-PAGE. Means with different letters (a–b) differ significantly (*p* < 0.05).

ImageJ software was used to analyze the change in the gray value of each subunit in potato protein. As shown in Figure 6A,C, compared with the control, the contents of patatin and protease inhibitors of TU20/28/40, DU40/60, and MU40 kHz were reduced to approximately 65.6% and 56.2% (*p* < 0.05), respectively, under reducing conditions. As displayed in Figure 6B,D, compared with the control, the content of patatin and protease inhibitors decreased to approximately 34.6% and 46.8% (*p* < 0.05) under nonreducing conditions. This result suggested that the soluble aggregates induced by online US/T40/pHS treatment were made up of patatin and protein inhibitors. The addition of β-ME broke the disulfide bond of the soluble aggregates. The contents of patatin and protease inhibitors increased to approximately 90.0% and 21.3% compared with those in nonreducing samples.

#### 3.3.6. Zeta Potential

**Figure 7:**
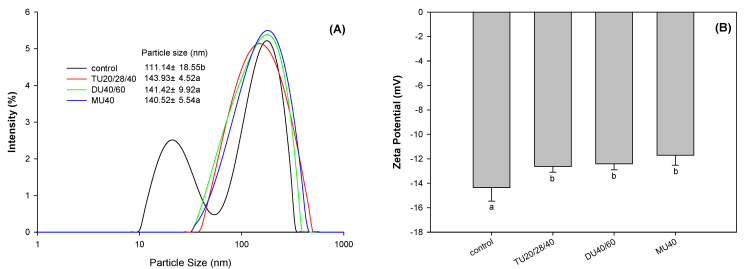
Effects of online-US/T40/pHS treatment at the frequency of TU20/28/40, DU40/60, and MU40 kHz on average particle size and particle size distribution (**A**) and zeta-potential (**B**) of potato protein. Means with different letters (a–b) differ significantly (*p* < 0.05).

The zeta potential of proteins can be used to characterize the surface charge of protein molecules. A change in protein structure has an effect on the amount of the protein surface charge [30]. As shown in Figure 7B, online US/T40/pHS treatment significantly changed the surface potential of potato protein (*p* < 0.05). Compared with the control, the surface potential absolute value of potato protein modified with TU20/28/40, DU40/60, and MU40 kHz decreased by 12.0%, 13.45%, and 18.4% (*p* < 0.05), respectively. The reduction in surface charge led to a weakening of the electrostatic repulsion between protein molecules, which was beneficial to promote the aggregation of proteins [31]. This finding was consistent with the particle size distribution results (Figure 6A).

### 3.4. Effect of Online Us/t40/pHS Treatment on the Morphology of the Potato Protein

**Figure 8:**
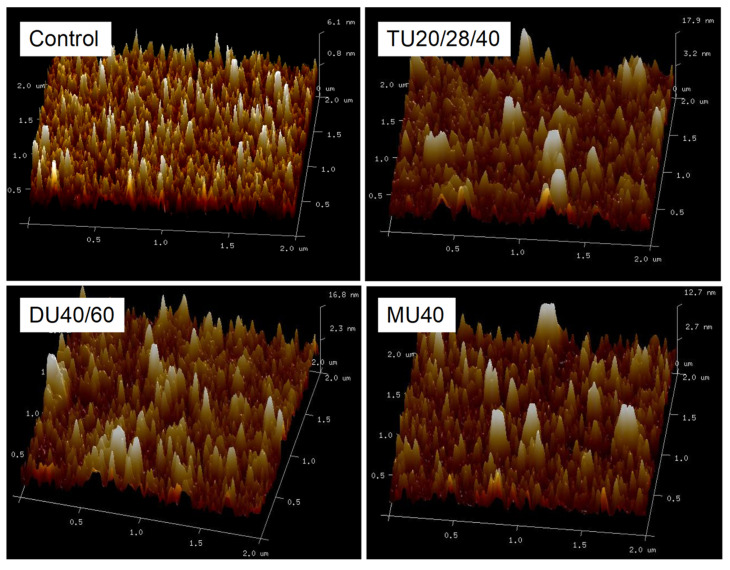
Atomic force microscopy (AFM) micrographs of potato protein treated with online-US/T40/pHS at the frequency of TU20/28/40, DU40/60, and MU40 kHz.

The aggregation of protein molecules induced by a physical or chemical modification can change the particle size of the protein particles. As shown in Figure 6A, the small peak at 10–50 nm disappeared, and the particle size distribution of potato protein indicated a right shift. Compared with the control, the average particle size of modified potato protein modified with TU20/28/40, DU40/60, and MU40 kHz increased by 29.5%, 27.2%, and 26.4% (*p* < 0.05), respectively. Furthermore, atomic force microscopy (AFM) was used to observe the morphology of potato protein treated with online US/T40/pHS. As displayed in Figure 8, potato protein without treatment was smaller than that with online US/T40/pHS treatment. This finding was consistent with the particle size results. Some large particles were displayed in the AFM images of the online US/T40/pHS-treated samples, which confirmed the SDS-PAGE results that indicated that large soluble protein aggregates in potato protein formed with online US/T40/pHS treatment. These aggregates might be due to the cavitation and mesothermal effects of ultrasound, as ultrasonic radiation promoted the unfolding of protein molecules under alkaline conditions. When the pH value was adjusted back to neutral, the proteins aggregated and eventually formed larger soluble aggregates.

## 4. Discussion

### 4.1. The Relationship between the Protein Structure and Solubility of Potato Protein

As shown in Table 3, the solubility of potato protein was positively correlated with the β-sheet content, λ_max_, FI, particle size, and zeta potential (*p* < 0.01), while the solubility of potato protein was negatively correlated with the α-helix, β-turn, and total sulfhydryl contents and H_0_ (*p* < 0.01). The intrinsic fluorescence results suggested that potato protein modified with online US/T40/pHS was partially unfolded. This finding was confirmed by the decrease in β-helix content. An unfolding of potato protein might expose tryptophan groups to a hydrophilic microenvironment [32]. The self-assembly of tryptophan groups driven by hydrophobic interactions might lead to the aggregation of potato protein. β-sheets easily exists in protein aggregates due to the effect of hydrogen bonds [33]. Therefore, the increase of the β-sheet content suggested that the unfolded protein molecules might form aggregates.

Otherwise, the covalent bonds formed in potato protein molecules might contribute greatly to the formation of protein aggregates. Our results showed that the online US/T40/pHS treatment decreased the total sulfhydryl content. These sulfhydryl groups might be oxidized to disulfide bonds, which can result in the formation of large molecular aggregates. The formation of aggregates was confirmed by SDS-PAGE. Disulfide bonds seemed to not be the only covalent bonds in these aggregates. Other types of covalent crosslinks were generated in aggregates, such as Schiff bases and tyrosine [10]. Interestingly, the large aggregates formed were soluble according to SDS-PAGE and AFM results. This might be because the online US/T40/pHS treatment exposed more hydrophilic groups and decreased the surface hydrophobicity, which is consistent with the surface hydrophobicity results. More hydrophilic groups might enhance the interaction between the protein and water molecules [8,34], which may be beneficial for protein solubility. The research results of Jiang et al. [8] showed that the solubility and surface hydrophobicity of pea protein isolate treated with online US/pH treatment increased significantly, which was contrary to the results of this study. In addition to the different research objects, the different ultrasonic equipment also greatly affects the change of protein structure. Jiang et al. [8] treated pea protein with an energy-gathered ultrasonic device with mono frequency of 20 kHz. In this paper, we used a kind of divergent ultrasonic equipment with triple frequency. The ultrasonic energy distribution on the samples induced by divergent ultrasonic equipment is usually more homogeneous than that by energy-gathered ultrasonic device.

Based on our results, the structural changes in potato protein were supposed to follow three steps. First, when the pH was adjusted to 12.0, potato protein was partially unfolded because of the increasing surface charge during the pH adjustment. As a result of structure unfolding, internal hydrophobic groups and sulfhydryl groups in potato protein molecules were exposed. Second, when ultrasound and moderate temperature were incorporated into the pH shifting treatment, the ultrasonic cavitation and moderate temperature may have lowered the active energy, rapidly increasing the unfolding degree of potato protein, and leading to the oxidation of closed sulfhydryl groups and their conversion into disulfide bonds. In addition to disulfide bonds, some other covalent bonds such as dityrosine and Schiff base were also formed while maintaining the pH at 12.0 for 1 h. Covalent bonds could lead to the formation of large protein molecules. Third, when the pH was adjusted back to 7.0, the repulsive forces induced by the high surface charge might lower the interaction between protein molecules. The steric hindrance of large molecules might also reduce interactions between protein molecules, leading potato protein to be converted into an incompletely denatured molten-globule state and potato protein molecules to undergo a random and disordered aggregation behavior [11], ultimately resulting in the loose structure of aggregates. Since more hydrophobic groups were entrapped into the inside of these aggregates, the proportion of hydrophilic groups on the surface of the protein was increased, which was beneficial to improve the solubility of potato protein.

### 4.2. The Relationship between the Structure and Digestion of Potato Protein

It was demonstrated that the solubility of potato protein was positively correlated with its digestibility (*p* < 0.01). An increase in the solubility of potato protein was able to enhance the substrate concentration of digestive enzymes, which might lead to a higher reaction rate of digestion reactions. Our amino group release results confirmed that the potato protein treated with online US/T40/pHS exhibited a higher reaction rate constant of digestion (k) than the control.

Similar to the solubility results, the digestibility of potato protein was significantly positively correlated with the β-sheet content, λ_max_, FI, particle size, and zeta potential (*p* < 0.01), but it was negatively correlated with the α-helix, β-turn, and total sulfhydryl contents and H_0_ (*p* < 0.01). The increase in FI and decrease in the content of α-helices suggested the unfolding of potato protein treated with online US/T40/pHS. The unfolding of this structure may expose more enzyme contact sites, which made the association of potato protein and digestive enzymes easier [35]. This unfolded structure was able to improve the digestion of potato protein, as shown in our previous work. The decrease in H_0_ suggested that the ratio of hydrophilic groups on the surface of the potato protein increased, which might improve the interaction of hydrophilic groups between potato protein and digestive enzymes.

## 5. Conclusions

The solubility and digestibility of potato protein was significantly improved by online ultrasound-assisted pH shifting treatment (online-US/T40/pHS). Online ultrasound treatment made pH shifting treatment more efficient in enhancing the solubility of potato protein. Online US/T40/pHS treatment with a triple frequency of 20/28/40 kHz, dual frequency of 40/60 kHz, and mono frequency of 40 kHz at the same energy density input resulted in higher solubility of potato protein. Moreover, online US/T40/pHS treatment reduced the surface hydrophobicity and total sulfhydryl content of potato protein. Meanwhile, this treatment changed the secondary structure of the protein and the microenvironment of tryptophan. The structural changes during online US/T40/pHS treatment promoted the aggregation of protein molecules and the formation of soluble macromolecular aggregates. This study demonstrated that online US/T40/pHS treatment may be an effective way to improve the functional properties of potato protein.

## Figures and Tables

**Table 1:** Characteristic parameters of the first-order kinetic equation for the release of the amino group in potato protein during gastric and intestine digestion.

		Control	TU20/28/40	DU40/60	MU40
Gastric digestion	y_0_ (mmol/L)	0.24 ± 0.08	0.21 ± 0.08	0.18 ± 0.09	0.22 ± 0.09
	A (mmol/L)	6.2 ± 0.52 ^b^	6.96 ± 0.39 ^a^	6.88 ± 1.32 ^a^	7.07 ± 0.27 ^a^
	K (min^−1^)	0.39 ± 0.06 ^b^	0.47 ± 0.02 ^a^	0.48 ± 0.13 ^a^	0.45 ± 0.02 ^a^
	R^2^	0.95 ± 0.01	0.98 ± 0.02	0.98 ± 0.01	0.97 ± 0.01
Intestinal digestion	y_0_ (mmol/L)	9.44 ± 1.12	9.7 ± 0.45	9.42 ± 0.04	9.42 ± 0.04
	A (mmol/L)	4.76 ± 0.88 ^b^	5.56 ± 0.13 ^a^	5.68 ± 0.19 ^a^	5.68 ± 0.19 ^a^
	K (min^−1^)	0.14 ± 0.02 ^b^	0.178 ± 0.02 ^a^	0.18 ± 0.01 ^a^	0.18 ± 0.01 ^a^
	R^2^	0.97 ± 0.03	0.99 ± 0.01	0.99 ± 0.01	0.99 ± 0.01

Means in the same row with different letters (a–b) differ significantly (*p* < 0.05).

**Table 2:** Effects of online-US/T40/pHS treatment at the frequency of TU20/28/40, DU40/60, and MU40 kHz on secondary structure of potato protein.

	α-Helix (%)	β-Sheet (%)	β-Turn (%)	Random Coil (%)
control	18.35 ± 0.85 ^a^	27.15 ± 1.05 ^b^	22.60 ± 0.40 ^a^	31.45 ± 1.25 ^a^
TU20/28/40	8.35 ± 0.75 ^b^	38.05 ± 0.95 ^a^	21.95 ± 0.15 ^a,b^	31.70 ± 0.10 ^a^
DU40/60	8.55 ± 0.05 ^b^	37.90 ± 0.20 ^a^	21.25 ± 0.75 ^a,b^	32.00 ± 0.20 ^a^
MU40	7.95 ± 1.25 ^b^	36.60 ± 1.40 ^a^	22.05 ± 0.95 ^a,b^	33.45 ± 1.65 ^a^

Means in the same column with different letters (a–b) differ significantly (*p* < 0.05).

**Table 3:** Pearson correlation of potato protein structure and functional characteristics.

	Solubility	Digestibility	α-Helix	β-Sheet	β-Turn	Random	Total Sulfhydryl	Free Sulfhydryl	H_0_	λ_max_	FI	Particle Size
digestibility	0.951 **											
α-helix	−0.985 **	−0.927 **										
β-sheet	0.957 **	0.915 **	−0.957 **									
β-turn	−0.713 **	−0.713 **	0.692 *	−0.723 **								
random	0.402	0.387	−0.401	0.154	−0.125							
total sulfhydryl	−0.959 **	−0.930 **	0.960 **	−0.912 **	0.721 **	−0.459						
free sulfhydryl	0.523	0.476	−0.537	0.677 *	−0.288	−0.305	−0.523					
H_0_	−0.989 **	−0.976 **	0.976 **	−0.960 **	0.724 **	−0.384	0.964 **	−0.507				
λ_max_	0.995 **	0.959 **	−0.987 **	0.976 **	−0.705 *	0.351	−0.956 **	0.571	−0.993 **			
FI	0.980 **	0.950 **	−0.957 **	0.925 **	−0.769 **	0.469	−0.957 **	0.461	−0.978 **	0.974 **		
particle size	0.941 **	0.889 **	−0.915 **	0.915 **	−0.640 **	0.319	−0.853 **	0.524	−0.921 **	0.939 **	0.907 **	
zeta potential	0.777 **	0.723 **	−0.756 **	0.756 **	−0.698 **	0.204	−0.776 **	0.567	−0.752 **	0.769 **	0.789 **	0.744 **

‘−’ Negative correlation; ‘+’ Positive correlation; FI: Fluorescence intensity; H0: Surface hydrophobicity. ** Means significant at *p* < 0.01. * Means significant at *p* < 0.05.
